# Assessment of the intensity and attractiveness of physical exercise while playing table tennis in an immersive virtual environment depending on the game mode

**DOI:** 10.1186/s13102-024-00945-y

**Published:** 2024-07-17

**Authors:** Jacek Polechoński

**Affiliations:** Institute of Sport Sciences, Academy of Physical Education in Katowice, Katowice, Poland

**Keywords:** Virtual reality, Table tennis, Physical activity, Physical education, Exercise intensity, Satisfaction

## Abstract

**Background:**

It appears that active video games (AVGs) and training apps that allow for physical activity (PA) in immersive virtual reality (VR) may be useful for sports, health-enhancing PA, and physical education (PE). Therefore, research is needed to identify their potential.

**Objective:**

The study aimed to evaluate the intensity and attractiveness of exercise during table tennis (TT) training in VR in arcade and simulation modes and to assess the potential for using such exercises in health-enhancing PA, sport, and PE.

**Methods:**

The research used the Racket Fury: Table Tennis VR. Exercise intensity during TT training in VR was evaluated by heart rate (HR) monitoring and rating of perceived exertion (RPE 6–20). The effectiveness of short-term TT training in VR was estimated based on the user’s performance in playing against an opponent with artificial intelligence (AI), satisfaction with playing TT was measured using the Physical Activity Enjoyment Scale (PACES), and the potential usefulness of the tested app in PA, sport, and PE was assessed based on a questionnaire for participating PE teachers (30 participants).

**Results:**

PA intensity during TT training in VR expressed as a percentage of maximum heart rate (HR_max_) was moderate but was significantly (*p* < 0.001; d=-0.830) higher in the easier arcade mode (69.50 ± 12.58%HR_max_) than in the simulation mode (64.10 ± 9.67%HR_max_). Despite the greater fatigue of respondents, user satisfaction was significantly higher in arcade mode. Users’ performance when playing with AI was significantly better after 20 min of training in VR than before training. PE teachers recognize the great potential of the app.

**Conclusions:**

The application tested is characterized by a beneficial PA intensity, with its level depending on the game mode. Facilitating strokes during a game of virtual TT promotes increased intensity of exercise and increased enjoyment of the PA. Short-term TT training in VR improves playing skills in a virtual environment. PE teachers spoke highly of the app and recognized the potential for using VR technology in PA, sports, and school PE.

## Background

Immersive virtual reality (VR), in which users are cut off from real visual external stimuli and instead can observe artificially created images and sounds and even tactile sensations, and move and interact with virtual objects, is used in various areas of human life. It is increasingly being used as a training tool [1]. VR technology is of great interest in situations where real-world training is difficult to organize, unsafe, or not very practical. Examples include training programs for soldiers [2], surgeons [3], paramedics [4], welders [5], and pilots [6]. There is also evidence that movement skills acquired in VR can be transferred to the real world [7–9], which may have important implications for sports training and recreational physical activity (PA). In sport and PA, VR is also increasingly being used to develop and diagnose motor abilities [10–14], practising various forms of exercise [15, 16], and even the assessment and analysis of certain movement parameters [17]. The first studies are being conducted on the effectiveness of VR exercises used in physical education (PE) classes in schools compared to typical forms of PA [18]. There are also reports that exercise in VR can be beneficial in the school PE process, resulting in improvements in health-related general physical fitness, cardiorespiratory endurance, muscular strength and endurance, power, and flexibility [19].

Given the above facts and being aware of the young generation’s fascination with information technology and knowing that users rate their satisfaction with PA in a virtual environment highly [20, 21] it seems that the use of VR in the school PE process needs to be seriously considered. However, this should be preceded by further multifaceted research. Assessments of the potential for using VR in PE should be made first of all by experienced teachers of the subject based on their experience of exercising in VR. A review of the available literature shows that such studies are lacking. Among the aspects of exercises in VR, one should take into consideration the health-enhancing nature of such a form of PA, the possibility of using it to learn sports technique and develop motor abilities, and the attractiveness of training.

Among the many apps currently available that enable training in VR, there are some that would be more or less useful in PE classes in schools. Particularly useful can be those that can make training in a virtual environment more effective, more enjoyable, and easier to implement than the typical solutions used in conventional PE lessons at school. It seems that these conditions can now be met by, among other things, applications for virtual table tennis (TT) training [22]. Implementing this sport in PE lessons causes many problems to teachers. This is due to organizational reasons, such as the low availability of equipment in the facilities, the time-consuming nature of creating the conditions for exercises associated with transporting the tables from the warehouse to the gymnasium, and having to lay them out. The small number of people who can exercise at the same time is also a problem. Furthermore, the game of TT is technically difficult, characterised by a high complexity of movements, and requires the player to make quick decisions related to the type of strokes and the positioning of the racket, which can be difficult in the initial period and can promote discouragement in the student. The high speed of the ball and its rotation is also a challenge for beginner tennis players. For example, in the case of fast spin shots, the speed at which the ball spins is between 3,000 and 5,000 rpm in amateurs, while it can exceed 8,000 rpm during professional matches [23]. Therefore, the use of VR technology in TT training enables a range of solutions to potentially facilitate the teaching of technical skills and game play. These include, among others: slowing down the speed of the ball and making it easier to return during the initial learning period, easier visualisation of the movements taught at a slower pace so that the person learning to play can better imagine these movements, the ability to assess the correctness of the movement patterns performed using sensors in goggles and controllers, and, in the longer term, additional sensors, and haptic gloves and suits. In a virtual environment, there is also the possibility of repeating the same strokes over and over again since the ball can be continuously returned to the user by a virtual opponent with artificial intelligence (AI) to exactly the same place. There are currently several consumer apps that allow TT training. Research is also being undertaken to improve player interaction with the virtual environment and improve the authenticity of the simulation [24]. There are also promising research experiments on the transfer of skills and experience gained from TT training in VR to the real world [7, 8].

Recently, many studies have been conducted to evaluate various aspects of PA in VR. First of all, they concerned the intensity of physical exercise during AVGs [15, 16, 25–28] and the attractiveness of this type of exercise [21, 28–31]. However, there is a lack of research that evaluates these aspects of PA in relation to virtual TT. The aim of this research is to fill this gap. Therefore, the exploratory study was planned and carried out to evaluate the intensity and attractiveness of exercise during TT training in VR in arcade (easier) and simulation (harder) modes and to assess the potential for using such exercises in health-enhancing PA, sport, and PE. This study aim allowed the following research questions to be posed:

1. What is the exercise intensity depending on the TT training mode in VR and is it sufficient in the context of health-enhancing PA recommendations?
2. Do arcade and simulation modes determine the level of satisfaction of users with PA?
3. Does a brief training session of TT in VR affect the player’s skill level in playing against a virtual opponent with AI?
4. How do experienced PE teachers assess the potential usefulness of the application studied in PA, sport, and PE?

## Study participants

Thirty experienced PE teachers participated in the study, including 11 women (age 50.73 ± 3.04 years, body mass 62.91 ± 7.11 kg, body height 167.36 ± 5.22 cm, work experience 27.09 ± 3.65 years) and 19 men (age 51.00 ± 4.36 years, body mass 84.95 ± 11.46 kg, body height 174.37 ± 12.81 cm, work experience 23.79 ± 7.41 years). Volunteers for the study were recruited among staff at the Academy of Physical Education in Katowice and schools in Katowice, Poland. All teachers had a master’s degree in PE and nine teachers additionally had a PhD degree in PE. The study included middle-aged people (45–59 years old) with at least ten years of work experience, who were professionally active and declared good health. According to the exclusion criteria used, people with motion sickness, sensitivity to flashing lights, epileptic seizures, and balance disorders were excluded from the study. The ability to play TT was also an inclusion criterion. All participants in the study declared experience of playing TT. They rated their technical skills at an average of 4.87 ± 1.66 points on a 10-point scale. None of the participants had previously used the app studied and had experienced TT training in VR. Participants also declared that they did not regularly use VR technology. Before the study, teachers were familiarized with the purpose and conduct of the study and the safety rules. They were also informed that they could opt out of participating in the study at any time. They also completed a personal questionnaire and signed a consent to participate in the study and to process their personal data. The study was conducted according to the guidelines of the Declaration of Helsinki and reviewed and approved by the Research Ethics Committee of the Academy of Physical Education in Katowice (protocol 9/2018, annex KB/27/2022). The research was carried out at the Jerzy Kukuczka Academy of Physical Education in Katowice, Poland, at a certified Laboratory of Research on Pro-Health Physical Activity (PN-EN ISO 9001:2015, certificate validity: 7.12.2021–16.12.2024).

## Methods

For VR immersion, we used the Oculus Quest 2 VR headset (Meta, CA, USA) consisting of VR glasses and two controllers. The training program was based on the use of the Racket Fury: Table Tennis VR app (Pixel Edge Games, Poland). It offers the possibility to compete in a multiplayer or offline options with a virtual avatar in the form of a hovering robot, consisting of a torso, arms, and head. Users can choose between arcade and simulation modes. In the arcade mode, the game’s mechanics make it easier for the player to perform the strokes and hit the table. In the simulation version, the physics of the game are very similar to those of a real-life TT game, requiring more skill from the player. The game is operated with 2 controllers. One is made visible in VR to the user as a virtual racket, the other is used to interact with menus and generate a virtual ball by pressing the trigger of the controller. In addition to competition, the app also allows training with a virtual opponent (practice option) who makes no mistakes (hits every ball) and whose parameters can be modified. The controllers are tracked with a high degree of precision, allowing accurate ball control. There are also various sounds associated with hitting the ball with the racket and bouncing on the table, depending on the strength of the impact, and vibrations with each stroke, which enhance the ball feel.

**Fig. 1:**
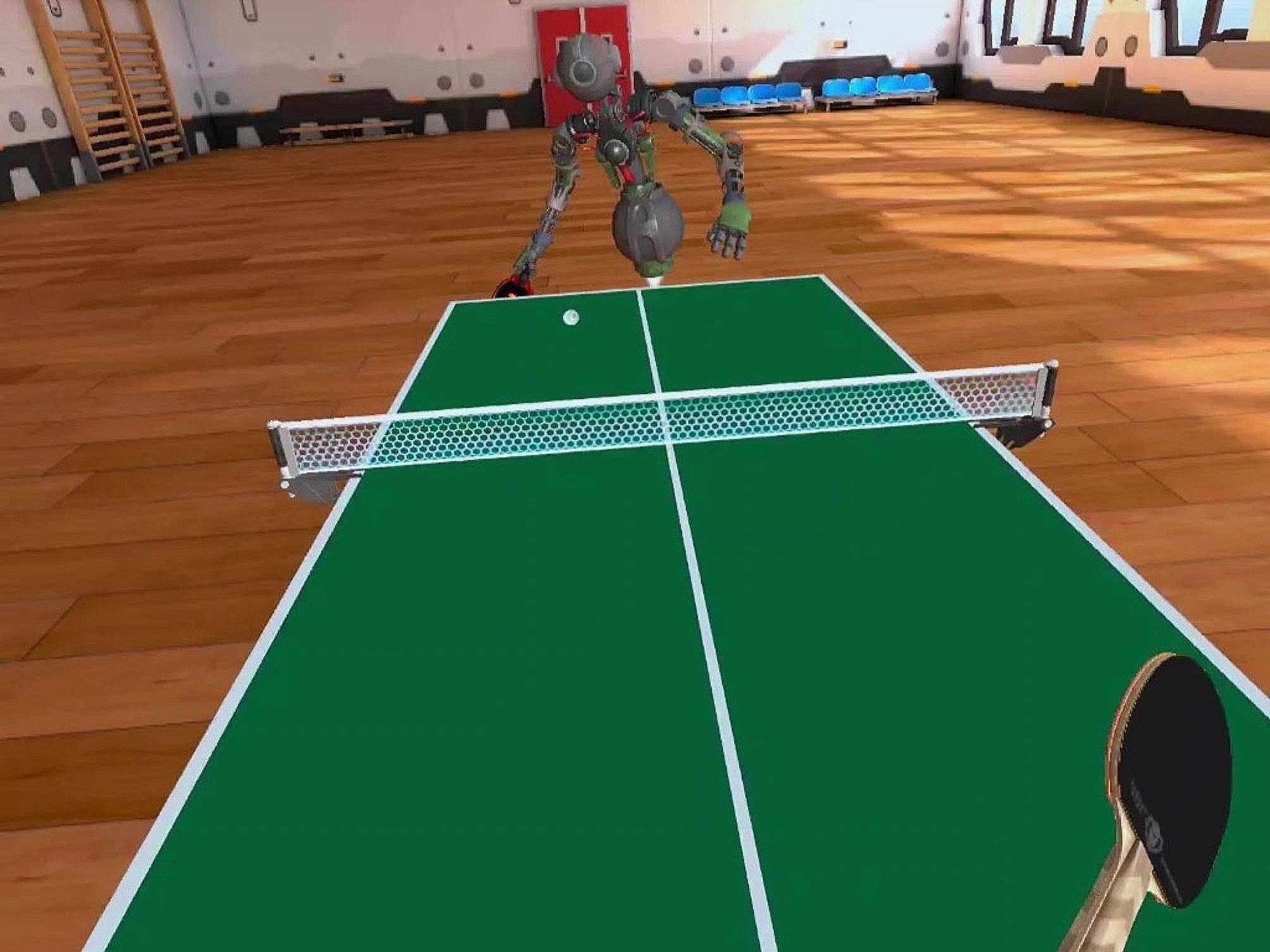
Game environment of Racket Fury: Table Tennis VR from the user’s perspective (screenshot)

**Fig. 2:**
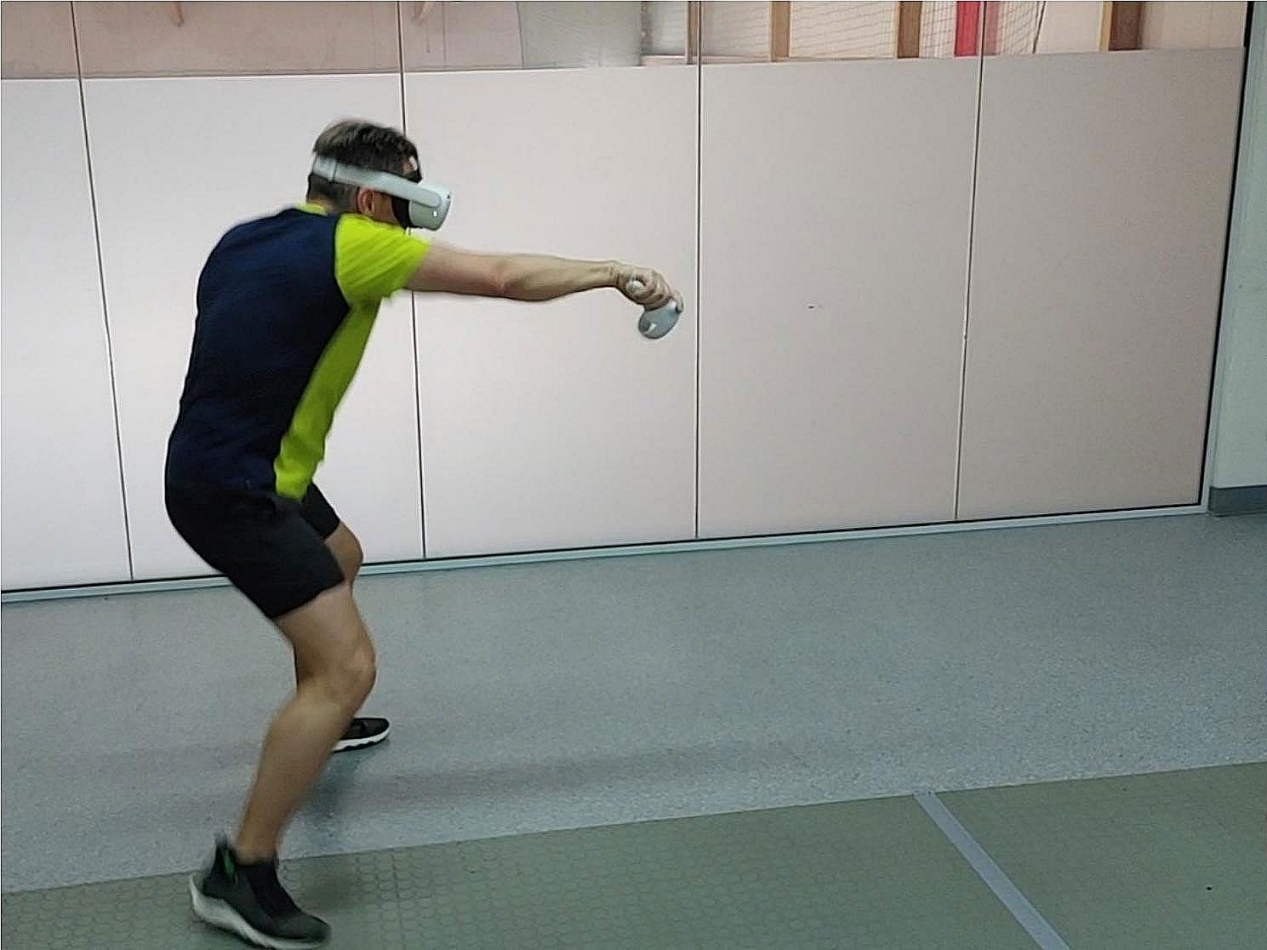
Participant playing table tennis with a virtual opponent

**Fig. 3:**
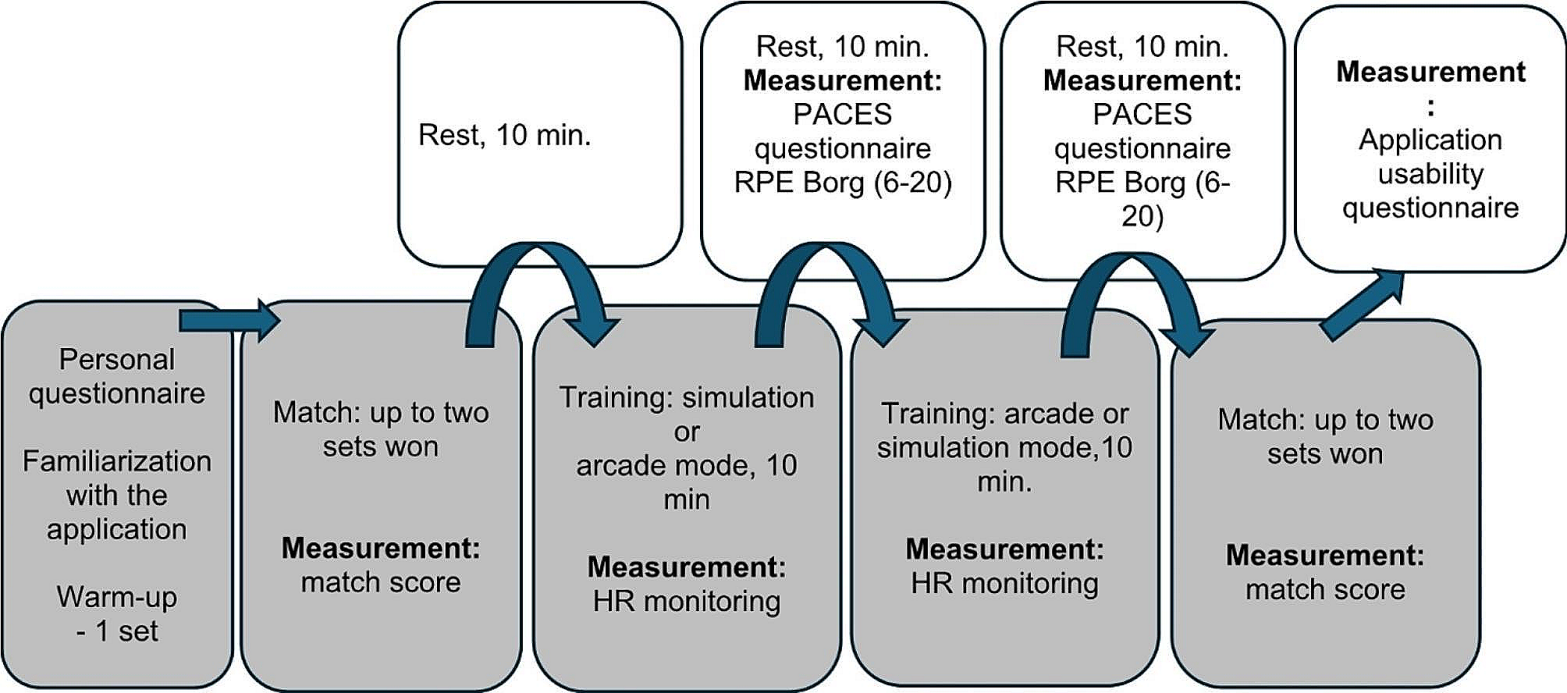
Diagram of research procedure

Before the study, participants were familiarized with the app and, as a warm-up and pre-adaptation to the VR environment, they played one set (up to 11 points) against a virtual opponent (AI in the form of a robot named Zen Jet) in the simulation mode, at the easiest difficulty level (Rusty Chalenge) with the app’s default/standard settings. They then took part, using the same mode, in a TT match to two sets won. After the game, the results were recorded. After a rest of 10 min, the participants performed two 10-minute workouts (practice option), also separated by a 10-minute rest, which consisted of hitting the ball with a virtual instructor (a Zen Jet robot) who made no mistakes. Workouts took place at default/standard settings, with one person starting with the arcade mode and another with the simulation mode, and then alternating the order. During each session, the participants’ heart rates were monitored using a Vantage V HR monitor (Polar Electro Oy, Kempele, Finland) coupled with a chest strap (Polar H10). The aim of this study was to calculate the average exercise heart rate (HR_ave_), from which PA intensity was later estimated based on the average percentage of maximum heart rate (%HR_max_). HR_max_ was previously calculated from the formula 208 − 0.7× age [32] and the results were related to PA intensity standards of the American College of Sports Medicine (ACSM) [33]. During the rest after each training session, participants completed a questionnaire about their satisfaction with the PA and subjectively estimated exercise intensity. Satisfaction with PA during training in VR was assessed using the Physical Activity Enjoyment Scale (PACES, a short version) consisting of 8 questions [34], which were answered after each test on a 7-point Likert scale. The result was an average calculated from all responses. Subjective feelings of fatigue from TT training in VR were assessed using the rating of perceived exertion (RPE) by Borg (6–20) [35, 36]. On this scale, a score of 10 to 11 indicates low-intensity exercise, a range of 12 to 13 indicates moderate intensity, and a score of 14–16 indicates high intensity [37]. After the training sessions, the study participants repeated the match against a virtual opponent until two sets were won. The parameters of the game were the same as in the first match, which was played at the beginning of the research. After its completion, the result was recorded. An illustration of the game environment is shown in Fig. 1, and the research stand and research participant are presented in Fig. 2. Finally, a self-administered questionnaire was used to collect the respondents’ opinions on the app and its usefulness in training and school PE. The questionnaire contained 15 items as shown in Table 1. The research procedure used is presented in the form of a diagram (Fig. 3).

### Statistical methods

Statistical calculations were performed using Statistica v.13 (TIBCO Software Inc., USA) and Jamovi v. 2.2.3.0 (Jamovi Project, Australia) software. Basic descriptive statistics such as arithmetic means, standard deviations, and structure indices were calculated. The Shapiro-Wilk test was employed to examine normality of the distribution. Verification of the significance of differences between the variables was carried out using either the parametric Student’s t test or the non-parametric Wicoxon test, depending on the data distribution. The level of statistical significance was set at α = 0.05. Effect size was estimated using Cohen’s d for the paired samples t-test and the rank-biserial correlation coefficient (r_rb_) for the Wilcoxon test. The Pearson’s linear correlation coefficient (r) was used as a measure of the correlation between objective and subjective measures of PA intensity.

## Results

### Exercise intensity during TT training in VR using racket fury: table tennis VR app depending on the game mode

**Fig. 4:**
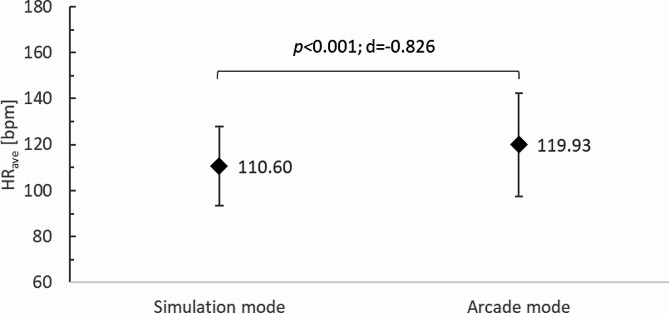
Mean heart rate (HR_ave_) of study participants according to TT training mode in VR; bpm, beats per minute; *p*, p-value; d, Cohen’s d value

**Fig. 5:**
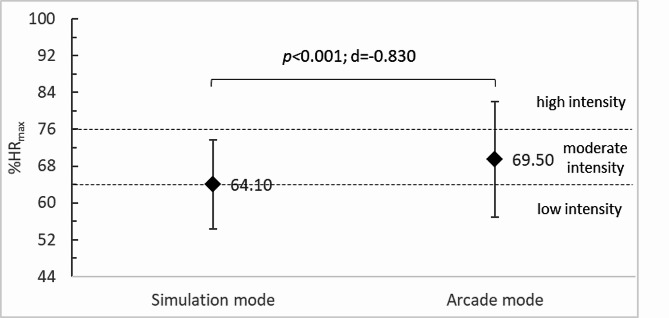
Exercise intensity of participants according to TT training mode in VR; HR_max_, maximum heart rate; *p*, p-value; d, Cohen’s d value

The HR_ave_ of study participants was significantly higher (*p <* 0.001; d=-0.826) for training in the arcade mode (119.93 ± 22.43 bpm) compared to the simulation mode (110.60 ± 17.33 bpm) (Fig. 4). Similar relationships were observed for exercise intensity. In the arcade mode, PA intensity was 69.50 ± 12.58%HR_max_ and was significantly higher (*p < 0*.001; d=-0.830) compared to that recorded in the simulation mode (64.10 ± 9.67%HR_max_). Given the ACSM classification [33], it should be concluded that in both cases the exercise was within the range of moderate intensity (64–76%HR_max_), recommended for health benefits by the World Health Organisation [38] (Fig. 5).

**Fig. 6:**
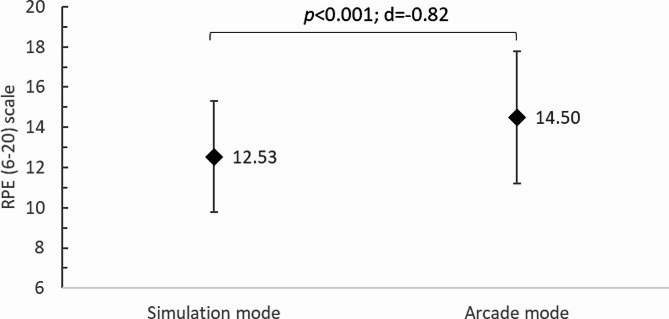
Rating of perceived exertion (RPE) according to TT training mode in VR; *p*, p-value; d, Cohen’s d value

Study participants also self-rated fatigue after each training session on the RPE scale (6–20). After training in the simulation mode, users perceived the intensity of their exercise to be 12.53 ± 2.76 points, while after training in arcade mode, they rated it at 14.50 ± 3.28 points. The difference found was statistically significant (*p* < 0.001; d=-0.82) (Fig. 6). Comparison of RPE reported by the participants with the classification of PA intensity [37] revealed that the teachers rated the PA in the simulation mode as moderate intensity, which was similar to that obtained from the objective measurement using HR monitor. Furthermore, physical exercise performed in the arcade mode was rated as vigorous by the participants, i.e. in this case the teachers overestimated its intensity in relation to objective measurements.

Pearson’s correlation analysis between objective and subjective measures of PA intensity showed a statistically significant positive relationship between intensity ratings estimated from %HRmax and Gunnar Borg’s RPE (6–20) scale for TT training in VR in both simulation (*r* = 0.513; *p <* 0.01) and arcade (*r* = 0.685; *p <* 0.001) modes. According to the scale proposed by [39], the correlation can be described as moderate in the first case and high in the second.

### PE teachers’ satisfaction with TT training in VR according to game mode

**Fig. 7:**
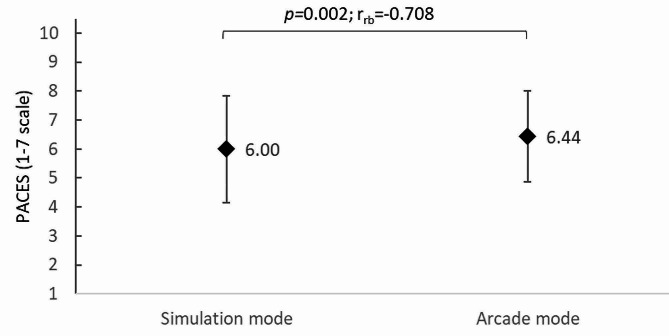
User satisfaction with TT training in VR according to game mode; PACES, Physical Activity Enjoyment Scale; *p*, p-value: r_rb_, rank-biserial correlation coefficient

The PACES survey of PE teachers (scale of 1 to 7) showed that teachers rated their satisfaction with TT training in VR highly in both simulation (6.00 ± 1.00 points) and arcade (6.44 ± 0.86 points) modes. Nevertheless, the easier (arcade) mode was rated statistically significantly better*(p =* 0.002; r_rb_=-0.708) (Fig. 7).

### Effect of short-term TT training in VR on the user’s level of skill in playing against a virtual opponent

**Fig. 8:**
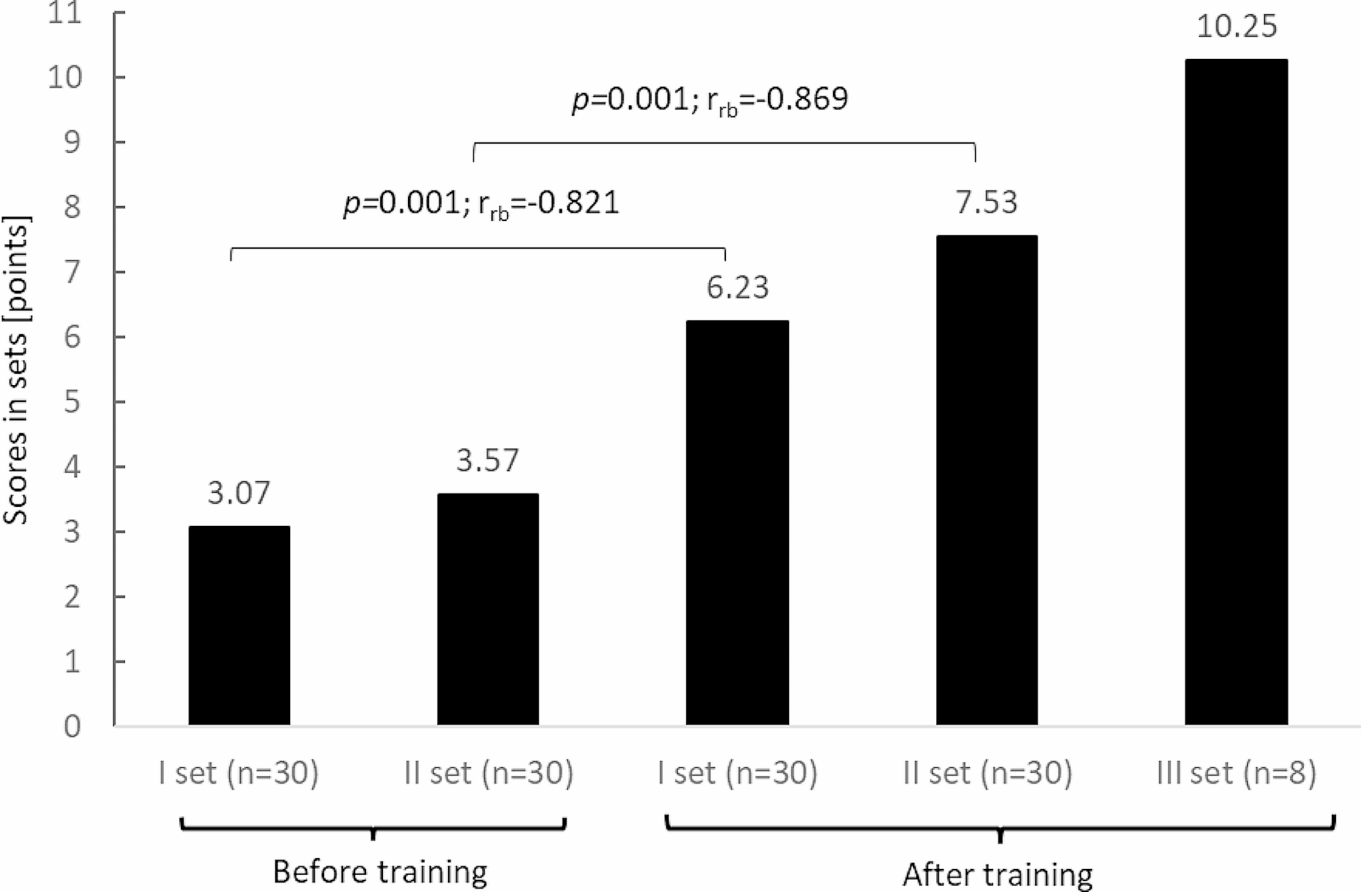
Users’ pre-training and post-training performance in playing TT with AI in VR; *p*, p-value: r_rb_, rank-biserial correlation coefficient

Before TT training in VR, only two participants had managed to win a match against a virtual opponent. Both matches were won in two sets. The other users did not win any set. After two ten-minute training sessions in VR, study participants won ten matches, including four times in two sets and six times in three sets, and 12 teachers won at least one set. In the subsequent sets, the participants achieved increasingly better results. The mean values are presented in Fig. 8. It was found that the results obtained after training in VR were significantly better than before training. In the first set of the first match, the average score was 3.07 ± 3.04 points and was significantly worse (*p =* 0.001; r_rb_=0.821) than that recorded in the first set of the second match (6.23 ± 3.56 points). Similarly, clear differences (*p =* 0.001; r_rb_=-0.869) occurred between the second set of the first match, where the average score was 3.57 ± 2.93 and the second set of the match played after TT training in VR, during which the teachers scored an average of 7.53 ± 3.58 points. The highest scores were recorded in the third set of the second match (10.25 ± 3.24 points), which was played by eight study participants (Fig. 8).

### EP teachers’ views on the potential usefulness of TT training in VR in PA and PE

Responses to a survey of PE teachers on their opinions of the app and its potential usefulness in sports training and the school PE show that respondents have a positive perception of Racket Fury: Table Tennis VR. Almost all (93%) claimed they would be willing to practise TT in VR provided they had the right hardware and software. The same percentage of teachers (93%) would recommend others to practise TT in VR. Only 7% had no opinion on these issues. However, a relatively small percentage of teachers (27%) felt that practising Racket Fury was more enjoyable than typical TT training in the real world. The majority of respondents (59%) disagreed with this statement and some (13%) did not have a clear position on this issue. Furthermore, all teachers were in agreement that practising TT in VR can offer health benefits and can be a supplement to a person’s leisure-time PA. Almost all respondents (90%) expressed the belief that the app they tested could be a useful training tool. Only one person (3%) disagreed with this opinion and two (7%) did not make a clear statement. With the question “Do you think the Racket Fury app could be a useful training tool?“, teachers were asked to justify their position. Those answering in the affirmative most frequently cited arguments such as that the application develops skills and motor abilities, motivates to train, allows the game to be played in a small space, anywhere and without specialised equipment, guarantees repetition, adds variety to the activity, allows the difficulty to be adjusted to the user’s ability, can be useful for people who have difficulty functioning in a group, and allows them to train independently. All of respondents also believed that the use of the Racket Fury app could help improve users’ motor skills. PE teachers were also of the opinion that the use of the Racket Fury app could help improve the technique of TT players practising the sport at different skill levels. Almost all respondents (90%) were convinced that the app could be useful in this respect with beginner athletes, while its usefulness among intermediate athletes was indicated by 77% of respondents. A considerable percentage of teachers (40%) even see the potential of Racket Fury in training the playing technique of advanced TT players. Another question concerned the possible use of the multiplayer mode of the app in inter-school competitions. This idea was welcomed by all the teachers surveyed. Also, all respondents stated that playing AVRGs such as Racket Fury in the multiplayer mode (described as active VR sport) could be a beneficial alternative to sedentary e-sports. Subsequent responses from respondents showed that almost all PE teachers had a negative perception of e-sports, as 96% of them did not think this form of leisure-time activity should be promoted. A completely different opinion was held by the respondents with regard to active VR sports, as they all believed that this form of activity should be promoted. Also, all interviewed teachers declared that if they had the appropriate equipment and software for active VR sport, they would use this form of PA in PE lessons or extracurricular activities (Table 1).

**Table 1:** Feedback from PE teachers about racket fury: table tennis VR app and its potential usefulness in PA and PE

Questions	Strongly disagree		Disagree		Somewhat disagree		Neither agree nor disagree		Somewhat agree		Agree		Strongly agree	
	*N*	%	*n*	%	*n*	%	*n*	%	*n*	%	*n*	%	*n*	%
1. Would you be involved in TT in VR if you had the right hardware and software?	0	0	0	0	0	0	2	7	9	30	7	23	12	40
2. Would you recommend practicing TT in VR to others?	0	0	0	0	0	0	2	7	5	17	8	27	15	50
3. Do you think that practicing Racket Fury is more enjoyable than typical TT training in the real world?	1	3	4	13	13	43	4	13	2	7	5	17	1	3
4. Do you think practicing TT in VR can have health-enhancing benefits?	0	0	0	0	0	0	0	0	5	17	2	7	23	77
5. Do you think practicing TT in VR can complement a person’s leisure-time PA?	0	0	0	0	0	0	0	0	1	3	7	23	22	73
6. Do you think the Racket Fury app could be a useful training tool?	0	0	1	3	0	0	2	7	2	7	16	53	9	30
7. Do you think that using the Racket Fury app can help improve the user’s motor skills?	0	0	0	0	0	0	0	0	5	17	10	33	15	50
8. Do you think that using the Racket Fury app can help improve TT technique in beginner players?	0	0	0	0	1	3	2	7	4	13	9	30	14	47
9. Do you think that using the Racket Fury app can help improve TT technique in intermediate players?	0	0	0	0	3	10	4	13	3	10	17	57	3	10
10. Do you think that using the Racket Fury app can help improve TT technique in advanced players?	2	7	0	0	5	17	11	37	3	10	5	17	4	13
11. Do you think playing the Racket Fury app in the multiplayer mode could be used in inter-school competitions?	0	0	0	0	0	0	0	0	3	10	8	27	19	63
12. In your opinion, could playing AVRGs such as Racket Fury in multiplayer mode (active VR sport) be a beneficial alternative to sedentary e-sports?	0	0	0	0	0	0	0	0	0	0	5	17	25	83
13. Do you think e-sports should be promoted?	13	43	7	23	9	30	1	3	0	0	0	0	0	0
14. Do you think active VR sports should be promoted?	0	0	0	0	0	0	0	0	3	10	5	17	22	73
15. If, as a teacher, you had the right equipment and software for active VR sport, would you use it in PE classes or extra-curricular activities?	0	0	0	0	0	0	0	0	3	10	6	20	21	70

## Discussion

According to objective tests (HR monitoring), the participants’ exercise intensity during virtual TT training in both arcade and simulation modes was at a moderate level. Therefore, it can be concluded that practicing TT in VR should offer users health benefits, provided regular training is undertaken as WHO classifies moderate-intensity PA exercise as health-enhancing [38]. The subjective perceptions of the participants assessed on the Borg’s RPE (6–20) scale correlated significantly with the objective measurements, although the teachers perceived exercise as intense in the arcade mode and moderate in the simulation mode. In terms of PA intensity, the results of the present study are in line with those of previous studies. Previous authors evaluating PA in VR during various forms of exercise also found moderate to even high-intensity exercise and indicated its health-enhancing nature [16, 40–43]. However, a review of the available literature shows that the level of intensity of virtual TT training has not been assessed to date. However, when comparing the intensity of PA while playing virtual and real TT based on the HR_avg_, it can be concluded that both efforts are similar. According to a systematic review that analyzed the physiological demands of various racket sports, HR_avg_ values ​​while playing TT range between 103.9 ± 15.09 and 146 ± 5.9 bpm [44]. The authors reached these conclusions after analyzing the results of seven studies [45–51].

The significantly higher exercise intensity found in the easier arcade mode compared to the more realistic and more difficult simulation mode in our study is puzzling. It is difficult to compare this with other studies as there are no studies in which the authors evaluated the intensity of VR exercise according to the level of realism of the application. Leaving aside the sense of realism, it is possible to analyse the results obtained in the context of the difficulty of the exercises, assuming that arcade mode is easier than simulation mode. Studies by Naugle et al. [41] showed that a higher level of difficulty of movement tasks in VR favours an increase in exercise intensity, which seems logical. The authors evaluated an AVRG called Holopoint in which participants used controllers as a bow and arrow to hit targets. When a target was hit, players had to dodge the projectiles that were thrown in their direction. The number of targets and the speed at which they appeared increased with higher levels. However, an inverse relationship emerged from the present research. The easier training mode (arcade) caused users to exercise at a higher intensity than the more demanding simulation mode, as confirmed by both objective and subjective measurements. This can be explained by the nature of playing TT, which is perceived as a technically difficult form of PA. The facilitation of strokes in the arcade mode meant that it took longer for the ball to be exchanged between the user and the virtual tennis player, and therefore there were fewer interruptions in the game that would reduce the intensity of PA. In the simulation mode, there were more breaks and ball exchange time was shorter. This observation may have important implications for the development of VR and AVRG training applications such as Racket Fury based on technically challenging movement activities. Indeed, the implementation of the arcade mode in these cases may contribute to increasing the exercise intensity in VR, and thus intensify its health-enhancing nature. The game mechanics characteristic of the arcade mode, which facilitate the use of the application, may also be useful for people who are less physically fit, especially those with reduced motor coordination. Forms of movement that are difficult to perform may discourage users from exercising, so in the initial phase of training, methods that make it easier to perform movement tasks should be used.

Study participants rated their satisfaction with TT training in VR highly, which is consistent with research by other authors assessing the attractiveness of various forms of exercise in a virtual environment [15, 16, 20, 21, 26, 27, 41, 42]. There are even reports that PA in VR can induce greater flow than a similar form of training in the real world [21]. When analysing the results obtained with the PACES, it is worth noting that the teachers rated their satisfaction with the easier form of TT training in VR (arcade mode) significantly higher compared to the more difficult simulation mode. This is probably due to the fact that the arcade mode favoured longer ball exchanges with the virtual tennis player and was probably better suited to the TT playing skills of the study participants. For the level of exercise intensity estimated for both game modes, it also appears that the higher participants’ satisfaction rating occurs for training characterized by higher exercise intensity and greater fatigue for the users. Facilitating training in VR through the introduction of an arcade mode can therefore both promote the intensity of exercise in a virtual environment and improve its appeal. This is undoubtedly a valuable tip for training application developers in VR and AVRGs. My findings may, to some extent, be confirmed by a study by Lemmens and von Münchhausen [52], which showed that matching users’ skills to the difficulty level of the popular rhythmic Beat Saber AVRG increases the perceived level of satisfaction and flow. On the other hand, a game option that is too difficult has the opposite effect. Frustration causes satisfaction and the level of perceived flow during PA to reduce. Interestingly, the overly easy form of the game did not lead to the expected decrease in satisfaction and flow as a result of boredom. A stronger sense of flow while playing the game was also associated with higher physiological arousal. Identification of the factors that influence satisfaction with exercise in VR can be helpful in developing software that users will enjoy using in the long term. It should be born in mind that satisfaction is an important motive for undertaking regular health-enhancing PA. How people feel during exercise determines their future training commitment [53]. The high level of satisfaction of the study participants could also result from the interaction with the controller, which when playing virtual TT is very realistic and resembles using a typical racket. In addition, when the ball contacts the virtual racket, there is a vibration that allows you to feel the ball, which gives you the feeling of a real game. It should also be borne in mind that the high rating of the attractiveness of virtual training may have been influenced by the novelty of the technology. Therefore, it would be worth conducting a similar study in the future among people who use VR for a longer period of time.

An important aspect of the study was the assessment of the effect of short-term TT training in VR on the user’s level of playing skills against a virtual opponent. It was shown that a training session of just 20 min can significantly affect a user’s performance and score in a virtual match. This is likely to be because the user is quickly adapting to the VR environment and taking their skills to the next level in VR. Of course, research showing the effect of TT training in VR on real-life playing performance is of greater practical relevance and should be the subject of further research. The transfer of TT playing skills from VR to the real world was investigated by Michalski et al. [7]. The authors studied young adult TT athletes (experimental group) and non-athletes (control group) in VR using the Eleven: Table Tennis VR app. The study included qualitative (expert assessment) and quantitative (target reflections) assessments in the real world before and after a session of seven training sessions in VR with a total duration of 3.5 h. VR training significantly improved participants’ real-world TT performance compared to a non-athlete control group in both quantitative and qualitative assessments of skills. A similar study was carried out by another group of researchers [8] using a system they developed for TT training in VR. The study found that after several training sessions in a virtual environment, participants in the experimental group showed significant improvements in the quality of TT strokes in the real world, which was manifested in a significant increase in the ball speed and a decrease in the height of the balls hit over the net. No changes were observed in the control group that did not participate in TT training in VR. Given the rapid improvement of TT playing skills in VR found during our study and the possible transfer of skills acquired in the virtual environment to the real world found in the studies mentioned above, the potential use of VR technology in the teaching of movement skills should be taken seriously. This applies not only to TT but also to other sports.

PE teachers that participated in the survey expressed a positive attitude towards Racket Fury: Table Tennis VR app and indicated its potential usefulness in PA and PE in schools. Almost all of them expressed a desire to practise TT in VR or would recommend others to practise this form of PA, although the majority preferred typical TT training in the real world. Teachers, on the other hand, were convinced that practising TT in VR could provide health benefits, supplement leisure-time PA and improve users’ motor skills. It is worth noting that similar opinions on the aforementioned issues were also expressed by future PE teachers and promoters of health-enhancing PA, who evaluated another AVRG in our previous study [16]. The PE teachers were mostly convinced that the Racket Fury app could be a useful training tool, and that using it could improve TT technique, especially in beginner and intermediate TT players. They supported this view with a number of convincing arguments, which are presented in detail in the Results section. All teachers were in agreement that AVRGs in multiplayer mode should be promoted and used for inter-school sports competitions as part of what is known active VR sports, which is a beneficial alternative to the badly perceived sedentary e-sports [54]. It is also worth noting the declaration of all teachers that, given the right equipment base, they would use active VR sports in PE lessons or extra-curricular activities. Analysis of the results of the survey reveals that experienced PE teachers were very complimentary about the application studied and the possibility to use VR technology in training and PE in school. This is quite surprising given that the respondents declared that they had never played virtual TT and did not use VR on a daily basis. It is likely that the experience of VR training made a big impression on the teachers, and their positive feedback may herald the use of modern technologies such as VR in PA and PE in the near future, especially as VR has been undergoing rapid development in recent years.

Finally, it is worth mentioning other qualities of the Racket Fury: Table Tennis VR app, which were not highlighted by the PE teachers. The exclusion of the human factor can be considered beneficial in the initial phase of teaching TT skills. At the beginning of the TT learning process, athletes often do not know how to hit the ball well, making it extremely difficult to exchange the ball with an opponent during their first attempts to play with athletes of similar skill, which can lead to frustration and discontinuation of training. By contrast, playing against a virtual opponent who is not wrong and can return every ball, does not complain, does not get tired, and is patient, the user can train comfortably, without stress or fear of criticism. If, on the other hand, someone prefers to play against a real opponent, they can use the multiplayer mode and train or play against opponents from all over the world.

## Limitations

Undoubtedly, the present study has several limitations. Among them, one can point to the method of assessing intensity of PA, which was based on HR monitoring. Optimally, a more precise indirect calorimetry method could be used for this purpose. HR monitoring was chosen because the study involved assessing different aspects of exercise in VR. Not only was an objective measurement of PA intensity made, but the severity of perceived exertion was also assessed. The research also looked at the effectiveness of training in VR, its attractiveness and the respondents’ opinions on the usefulness of this form of exercise in health-enhancing PA and PE. Therefore, there was a risk that the masks used in calorimetry could affect the subjective assessment of the participants as wearing them is unpleasant and may even cause considerable discomfort in some people. While pointing out the limitations, one should also refer to the assessment of the effects of short-term TT training in VR on performance in playing against a virtual opponent. Research into the transfer of skills acquired in VR to the real world would undoubtedly be more valuable. However, this would require the development of a suitable tool to assess playing skills in a real-world environment, conducting several training sessions and examining a control group. Therefore, based on the results of our study, we should rather infer the user’s ability to adapt quickly to the VR environment (which increased performance in playing with AI) rather than a significant improvement in playing skills. Furthermore, the use of the questionnaire designed by the authors in the study can also be considered a limitation, as it may make it difficult to compare the results with those of other authors. It should be mentioned, however, that the questionnaire was only an additional element of the present study and no similar questionnaires have been used in previous studies in which PE teachers would comment on AVGs and their potential usefulness in PA or PE. Therefore, the responses of the research participants can be considered valuable. Due to the fact that virtual TT was a new experience for users, it should be borne in mind that the obtained measurement results regarding exercise intensity and their attractiveness may have been influenced by the newness of the technology. Therefore, further research should be conducted among users who systematically use VR.

## Conclusions

In conclusion, the Racket Fury application used in VR is characterized by a moderate (i.e., beneficial) exercise intensity, with its level depending on the playing mode. In the easier (arcade) mode, users train more intensively and experience more fatigue than in the more difficult (simulation) mode, but they feel more satisfaction with PA. Therefore, facilitating strokes during a game of virtual TT promotes increased intensity of exercise and makes exercise more attractive. Short-term TT training in VR improves the ability to play in a virtual environment, which manifests itself in better results obtained by users during matches against AI. This is probably a manifestation of the rapid adaptation of users to the virtual environment. PE teachers with many years of professional experience spoke highly of the app tested and recognized the potential for using VR technology in sports training and PE in school.

Based on the results of the present study and the previously cited studies by other authors, it can be assumed that exercise in VR will be increasingly used in health-enhancing PA and PE, as it is characterised by an intensity that guarantees health benefits, is attractive to users, and is gaining recognition from experts. Due to the rapid development of immersive information technologies, it can also be assumed that VR training will become an important supplement to conventional forms of PA in the near future, and it is not excluded that it will be an alternative solution.

## Data Availability

The datasets used and/or analysed during the current study are available from the corresponding author on reasonable request.

## Abbreviations

% HR_max_: Percentage of maximal heart rate
AI: Artificial intelligence
AVG: Active video game
AVRG: Active virtual reality game
HR_avg_: Average heart rate
HR_max_: Maximum heart rate
PA: Physical activity
PACES: Physical Activity Enjoyment Scale
PE: Physical education
RPE: Rating of Perceived Exertion
TT: Table tennis
VR: Virtual reality
WHO: World Health Organization
